# Opioid-induced constipation in internal medicine: recognition and management pathways

**DOI:** 10.1007/s11739-025-04091-2

**Published:** 2025-08-28

**Authors:** Piero Portincasa, Chiara Valentina Luglio, Silvia Sozzi, Claudia Conforti, Gianluca Bitonto, Michele Papavero, Gyorgy Baffy, Agostino Di Ciaula

**Affiliations:** 1 Division of Internal Medicine ‘A. Murri’ and Department of Precision and Regenerative Medicine and Ionian Area (DiMePre-J), University of Bari ‘Aldo Moro’, Bari, Italy; 2 Department of Medicine, VA Boston Healthcare System, Boston, MA USA; 3 Department of Medicine, Brigham and Women’s Hospital, Harvard Medical School, Boston, MA USA

**Keywords:** Bristol stool scale, Gut transit, Laxatives, Naldemedine, Polyethylene glycol, Rome IV criteria

## Abstract

Opioid-induced constipation (OIC) remains one of the most frequent and distressing gastrointestinal side effects encountered by patients on chronic opioid therapy. Despite the high prevalence, OIC is frequently underdiagnosed and inadequately managed, with critical effects on the quality of life of patients. Aim of this review is to promote the awareness about OIC in the context of internal medicine. We examined the statement EnhanCing Healthcare Outcomes in Opioid-Induced Constipation (ECHO OIC) by European experts to streamline the diagnosis and management of OIC in clinical practice. Such guidelines have been further discussed by an Italian expert consensus to provide national customization and a multidisciplinary approach. The key finding is the implementation of a multi-step clinical pathway for prevention, diagnosis, treatment, and long-term management of OIC, taking into account the improvement of quality of life of patients. In conclusion, we urge to expand awareness about OIC. The seven-step diagnostic–therapeutic pathway approach formulated by ECHO OIC is a pragmatic and scalable model to improve OIC management with emphasis on education, early intervention, monitoring, tailored pharmacologic strategies, and coordinated referral when necessary.

## Introduction

The widespread use of opioid analgesics in patients with cancer-related and non-cancer pain has expanded significantly worldwide, raising concerns about possible dependency and highlighting the importance of managing their adverse effects. In the United States, 4–5% of the population receives opioid prescriptions, primarily for non-cancer pain, but broader estimates suggest that this figure may be higher when accounting for non-medical use [1, 2]. Similarly, opioid consumption in the European Union surged by nearly 40% between the late 1990s and mid-2010s, particularly in Western and Northern Europe [3]. Although Italy reports comparatively lower opioid use, consumption has been steadily rising [3, 4].

Opioids are responsible for centrally mediated analgesia but they also bind to *μ*, *κ*, *δ* receptors ubiquitously expressed in the enteric nervous system (ENS) and, therefore, present in the gastrointestinal tract. In particular, *κ*-receptors are expressed in the stomach and small intestine, while μ-receptors are expressed in the small intestine and proximal colon [5]. Such wide receptor distribution explains the occurrence of opioid-induced bowel dysfunction from the esophagus to the anus, with opioid-induced constipation (OIC) being the most common complication [6]. Indeed, up to 80% of patients on chronic opioids report OIC [7–9] and have significant impairment of the quality of life (QoL) [10]. The pathophysiology of OIC is primarily due to the activation of enteric *µ*-opioid receptors, which reduces GI motility, enhances fluid absorption in the gut, and increases sphincter tone [11–13]. Unlike other side effects, such as sedation or nausea, constipation often persists with continued opioid use [14].

Despite its prevalence, OIC remains frequently underdiagnosed and inadequately treated [15–17]. Patient and physician awareness is limited, particularly among general practitioners [18]. According to European patient surveys, dissatisfaction with constipation treatment is common, and many patients question the trade-off between analgesia and GI symptoms [19]. Furthermore, an Italian study among opioid prescribers found that nearly 40% did not routinely assess bowel function, and most physicians lacked adequate training on OIC management [20]. A follow-up confirmed progress but also emphasized ongoing educational needs [21].

Current best practices recommend a combination of non-pharmacological and pharmacological interventions [22]. While diet, hydration, and exercise may be beneficial, they are often unfeasible for frail or elderly patients. First-line treatments typically involve laxatives, though their efficacy is often limited due to nonspecific mechanism of action. Newer targeted treatments, the peripherally acting *µ*-opioid receptor antagonists (PAMORAs), such as methylnaltrexone, naloxegol, and naldemedine, offer more pathophysiological precision in managing OIC [22–25]. Despite this background about guidelines [26, 27], PAMORAs are still used by a minority of opioid-prescribing physicians[20, 28]. A recent survey in a tertiary care center revealed that about 73% of patients diagnosed with OIC were eligible for a PAMORA but only the 28% of these patients received a prescription for it [28].

The EnhanCing Healthcare Outcomes in Opioid-Induced Constipation (ECHO OIC) initiative, developed by a European multidisciplinary committee, proposes practical frameworks for managing OIC across healthcare settings [26, 27, 29]. A further step was the refinement of guidelines at a national level to suit Italian clinical practices [30].

Here, we discuss the rational steps to prevention and management of OIC to guide both primary and secondary healthcare professionals. In this respect, we do believe that internists should be adequately trained about this important issue.

## Steps involved in the management of OIC

### Early patient education at opioid initiation

The culture of pain management has advanced significantly in recent years but awareness regarding OIC needs further educational improvement. Patients receiving effective pain relief through opioids may paradoxically experience a greater burden of symptoms from constipation, than from the underlying pain itself. This misalignment significantly impacts the QoL and often remains unaddressed in routine clinical care.

In addition, opioid-treated patients typically present with complex medical needs, and adverse effects such as constipation are frequently deprioritized with respect to the primary condition. The awareness of treatment-related side effects is relatively higher in oncology, where patients and clinicians are more accustomed to addressing therapy-induced complications. Conversely, patients with chronic noncancer pain, who comprise 40–60% of the chronic opioid-using population, often under-report OIC symptoms [31]. This issue is especially pronounced among elderly patients, who represent a substantial portion of the chronic pain demographic. In this group, constipation is frequently under-recognized or accepted as a natural consequence of aging and reduced gastrointestinal motility [32, 33].

It is, therefore, essential that physicians, at the time of opioid prescription, proactively inform patients that constipation is a near-universal side effect of opioid therapy [34]. Moreover, patients should be educated that constipation encompasses not only reduced stool frequency but also symptoms such as bloating, straining, hard stool consistency, incomplete evacuation, and abdominal discomfort.

Nurses play a pivotal role in patient education, often engaging in longer and more open conversations that facilitate better understanding. Comprehensive and early education is key to preventing patients from independently reducing or discontinuing their opioid therapy, which occurs in about 20–30% of cases [35]. This discontinuation can compromise pain control, decrease health-related QoL, and increase healthcare resource utilization.

### Initiate prophylactic laxatives

For many patients—particularly the elderly, those with advanced cancer, or individuals living with chronic pain—lifestyle modifications such as increased physical activity or dietary changes are often impractical or unfeasible. Moreover, interventions such as enemas or manual evacuation techniques are not only distressing but may aggravate symptoms and consequences of constipation. Similarly, unsupervised use of over-the-counter remedies or non-standard home treatments can lead to drug interactions or complicate the overall management of opioid-induced side effects.

Consistent with current international guidelines [22–25], it is strongly recommended to initiate laxative therapy as a preventive measure at the time opioids are prescribed for long-term use (i.e., strong strength of recommendation, moderate quality of evidence) [22]. Eventually, the decision to administer laxatives prophylactically or at the first signs of constipation should be individualized and discussed between clinician and patient, based on comorbidities, expected opioid duration, and prior bowel habits. Bowel function, including frequency of evacuation and stool form using validated tools, such as the Bristol Stool Form Scale [36], should be assessed at baseline and monitored throughout treatment.

Laxatives encompass a wide range of agents with varying mechanisms of action (stool softeners, lubricants, osmotic agents, and stimulants). Of note, not all agents are suitable for managing OIC. For instance, fiber-based bulking agents may worsen bloating and are generally contraindicated in OIC. Prokinetic agents also offer limited efficacy in this context.

Among the options, osmotic laxatives [37], and in particular polyethylene glycol (PEG)/macrogol, are regarded as the preferred first-line treatment. These agents are minimally absorbed in the gut and act by increasing stool water content, promoting softer and more regular bowel movements. Macrogol has a strong safety profile, remains pharmacologically inert, and reaches the colon intact. Its efficacy has been demonstrated in placebo-controlled trials [38]. Stimulant laxatives as bisacodyl, senna, and sodium picosulfate represent an alternative option, especially for short-term use or when macrogol alone is insufficient. While robust randomized controlled trials are lacking for these agents in OIC, they are supported by extensive clinical experience.

When constipation persists despite the use of appropriately selected and dosed laxatives, a condition termed laxative-refractory OIC is considered. The Agenzia Italiana del Farmaco (AIFA), in its Note 90, defines this state as a lack of response after three consecutive days of treatment [39]. Based on this criterion, it is recommended to clearly instruct patients to contact the physician if constipation persists after 3 days of laxative therapy, to allow for timely reassessment and possible escalation of treatment.

### Assess for OIC within 2 weeks

Available evidence shows that the management of OIC in Europe is inadequate not only in terms of treatment, but also in terms of initial assessment and following reassessment of patients [40].

Although laxatives are commonly prescribed to prevent or manage OIC, evidence suggests that they are often insufficient as a standalone intervention [41]. Therefore, close and systematic monitoring of bowel function is essential from the outset of opioid therapy.

OIC may emerge immediately after opioid initiation or develop gradually with continued use [42]. For this reason, it is recommended a first structured assessment within 2 weeks of starting opioid treatment. At this point, the diagnosis of OIC should be guided by the Rome IV criteria (Table 1), which emphasize not only stool frequency but also the presence of straining, hard stool consistency (Bristol Stool Form Scale 1–2), and a sense of incomplete evacuation [43–45]. These criteria help differentiate true OIC from other forms of constipation, and are particularly valuable for non-specialist clinicians.

**Table 1:** Rome IV diagnostic criteria for OIC [43]

1	New, or worsening, symptoms of constipation when initiating, changing, or increasing opioid therapy, that must include two or more of the following: I. Straining during more than ¼ (25%) of defecations II. Lumpy or hard stools (Bristol Stool Form Scale 1–2) more than ¼ (25%) of defecations III. Sensation of incomplete evacuation more than ¼ (25%) of defecations IV. Sensation of anorectal obstruction/blockage more than ¼ (25%) of defecations V. Manual maneuvers to facilitate more than ¼ (25%) of defecations (e.g., digital evacuation and support of the pelvic floor) VI. Fewer than three spontaneous bowel movements per week
2	Loose stools are rarely present without the use of laxatives

A detailed clinical history is essential to identify any additional or alternative causes of constipation. Physicians should inquire about the onset, progression, and duration of symptoms and assess baseline bowel habits. Tools as the Bristol Stool Form Scale can be used to standardize the evaluation of stool consistency and support ongoing monitoring [36].

To assess the severity and impact of constipation, particularly in cases, where laxatives appear ineffective, the use of the Bowel Function Index (BFI) is strongly recommended [46], since its use can generate clinically relevant improvement in the management of OIC [47]. The BFI is a validated, clinician-administered tool specifically developed for opioid-treated patients with pain. It captures three key dimensions of bowel function from the patient’s perspective, i.e., 1) ease of defecation; 2) sensation of incomplete evacuation, and 3) personal judgment of constipation. Each item is scored on a visual analogue scale (0 = not at all, 100 = very strong), reflecting symptom severity over the previous 7 days. Although the BFI has not yet been formally validated in Italian, it is a clinically feasible, easy to administer, and sufficiently sensitive tool to detect changes in OIC severity in the routine practice. In particular, a BFI score ≥ 30 may indicate inadequate response to first-line therapy and should prompt clinicians to consider treatment escalation, including evaluation for PAMORA initiation [48].

### Prescribe PAMORAs for laxative-refractory OIC

Given that OIC results from the direct agonist action of opioids on peripheral µ-opioid receptors within the enteric nervous system, the management of this condition should move beyond nonspecific symptom control when first-line laxative therapy fails. In such cases, PAMORAs offer a rational, targeted approach.

**Fig. 1:**
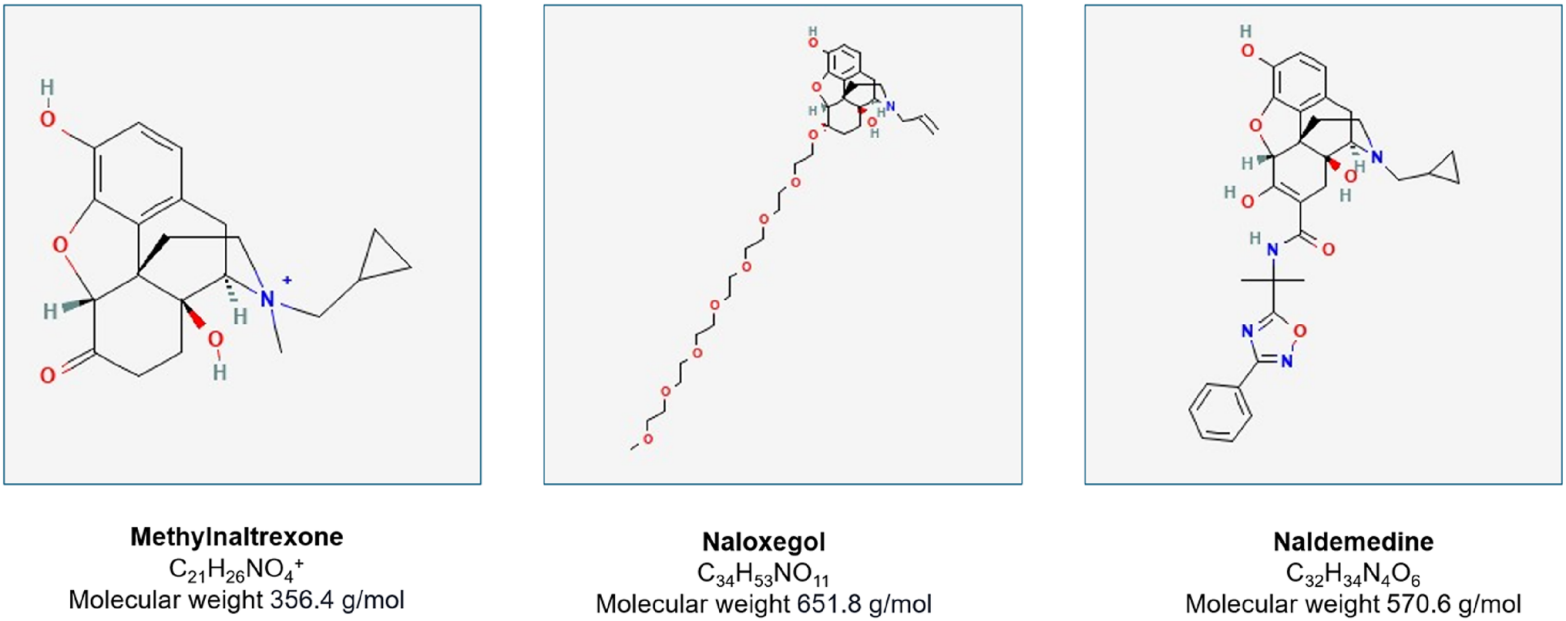
Chemical formulas of three PAMORAs: Methylnaltrexone, Naloxegol and Naldemedine

Currently, three PAMORAs are approved for the treatment of OIC, i.e., methylnaltrexone, naloxegol, and naldemedine (Fig. 1). These agents selectively block opioid receptors in the gastrointestinal tract, thereby restoring gut motility while preserving central analgesic efficacy. Their pharmacological properties—high polarity, large molecular size, and low lipid solubility—effectively prevent crossing of the blood–brain barrier at therapeutic doses [12, 49–51].

The American Gastroenterological Association (AGA) guidelines recommend PAMORAs for patients with laxative-refractory OIC, with naldemedine receiving the strongest endorsement based on evidence quality [22]. Notably, naldemedine antagonizes not only µ-receptors but also κ- and δ-opioid receptors, which are variably distributed across the gastrointestinal tract [52].

The clinical efficacy of naldemedine has been demonstrated in three pivotal randomized controlled trials: COMPOSE-1, COMPOSE-2, and COMPOSE-3 [53, 54]. COMPOSE-1 and -2 showed that naldemedine significantly increased the proportion of responders (i.e., patients achieving ≥ 3 spontaneous bowel movements per week) with a pooled risk ratio of 1.51 (95% CI: 1.32–1.72) versus placebo. COMPOSE-3, a 52-week trial, confirmed the long-term efficacy and safety of naldemedine, reporting sustained improvements in bowel movement frequency, symptom relief, and patient quality of life.

Besides efficacy, the use of naldemedine has been reported as safe also in vulnerable subjects as elderly [55]. The use of naldemedine induce positive effects on OIC also in pediatric patients, although the possible occurrence of grade 2 diarrhea indicates the need for further studies to determine the optimal dose of this drug in children[56].

The AGA also strongly recommends methylnaltrexone and naloxegol, albeit with conditional strength due to limitations in the supporting evidence[22]. Methylnaltrexone has been evaluated in multiple RCTs [57–61], though not all adhered to the FDA primary outcome standard of ≥ 3 rescue-free bowel movements per week. Despite demonstrating significant improvements in bowel function, the evidence was rated as low quality due to inconsistencies, indirectness, and imprecision across outcomes [22]. Nonetheless, its subcutaneous formulation is considered advantageous in select clinical scenarios, such as in palliative care or patients with limited oral intake.

Naloxegol, an oral PAMORA, is supported by two RCTs [62, 63]; an open-label extension study [64], and a post hoc analysis [65]. Across these studies, naloxegol was significantly more effective than placebo, including a notable reduction in the median time to first spontaneous bowel movement. While naloxegol is also strongly recommended for laxative-refractory OIC, the evidence is considered moderate quality due to imprecise data estimates [22].

In summary, PAMORAs represent an evidence-based, mechanism-specific therapeutic option for patients with OIC who do not respond to conventional laxatives. Among them, naldemedine currently offers the most robust clinical evidence, while methylnaltrexone and naloxegol provide effective alternatives with distinct routes of administration and pharmacologic profiles that may inform individualized treatment decisions.

### Educate patients on PAMORA use and monitor interactions

When initiating treatment with a PAMORA, clinicians must take several precautions to ensure safe and effective use. These considerations include evaluating drug interactions, managing co-administered therapies, and providing thorough patient counselling.

- Drug interaction and pharmacokinetic considerations. PAMORAs are associated with clinically relevant pharmacokinetic and pharmacodynamic interactions, particularly involving cytochrome P450 (CYP450) enzymes and P-glycoprotein (P-gp) transporters. Both naloxegol and naldemedine are substrates of CYP3A4 and P-gp, and their plasma concentrations may be significantly affected by coadministration with strong CYP3A4 inhibitors or inducers, as well as P-gp modulators [66, 67]. Therefore, careful review of the Summary of Product Characteristics (SmPC) is essential prior to prescribing. In contrast, methylnaltrexone exhibits minimal metabolism via CYP enzymes and has limited potential for drug–drug interactions, making it a preferable option in patients with complex pharmacotherapy regimens or hepatic impairment [68]. All PAMORAs carry a warning regarding the risk of opioid withdrawal, particularly when used in combination with mixed opioid agonists/antagonists (e.g., buprenorphine and pentazocine) or fixed-dose opioid-antagonist combinations (e.g., morphine/naltrexone). These combinations may diminish analgesic efficacy or precipitate withdrawal symptoms, especially in patients taking full opioid agonists such as oxycodone [48].
- Concomitant laxative use. While PAMORAs are frequently prescribed following the failure of conventional laxatives, their simultaneous use with laxatives during initial therapy may increase the risk of diarrhoea, particularly in the early days of treatment. To mitigate this risk, the recommendation is withholding or reducing existing laxative therapy at the initiation of a PAMORA. Follow-up evaluation should determine the need for reintroducing laxatives based on individual symptom control and tolerability.
- Patient counseling and adherence. Patient education is critical to the success of PAMORA therapy. It is important to clearly explain that the medication must be taken regularly as prescribed, not on an as-needed basis; that self-discontinuation should be avoided, even if bowel function improves, unless directed by the healthcare provider; the potential for transient gastrointestinal symptoms, such as diarrhoea or cramping, particularly during the first days of treatment.

By fostering a clear understanding of the therapeutic goals and expected course, clinicians can improve patient adherence and reduce unnecessary discontinuation of therapy.

### Monitoring the effectiveness and tolerability of PAMORA therapy

Once a PAMORA is initiated, it is essential to implement regular and structured follow-up to assess both therapeutic efficacy and potential adverse effects. It is recommended to perform an initial evaluation within 1 week of starting therapy, followed by monitoring at least every 2 weeks thereafter.

PAMORAs are generally well-tolerated, with the most commonly reported side effects, including abdominal pain, diarrhoea, and nausea. These events are typically mild to moderate in severity and tend to resolve without requiring discontinuation of treatment [65–67]. Notably, diarrhoea appears more frequently in patients receiving opioids for cancer-related pain, likely due to overlapping gastrointestinal vulnerabilities [51].

A standardized and validated assessment tool is essential to assess treatment response and to differentiate between therapeutic and adverse events. In this respect, the BFI is specifically designed for this purpose in opioid-treated populations. It offers a rapid and quantitative measure of bowel function from the patient’s perspective and is sensitive enough to detect clinically meaningful changes over time [46, 48]. The regular use of the BFI not only supports timely adjustments in therapy but also helps identify suboptimal responses or emerging tolerability issues, enabling individualized and proactive OIC management.

### Management of inadequate response to PAMORA therapy

If no clinically meaningful improvement in bowel function is observed after 4 weeks of treatment with a PAMORA, the therapeutic strategy should be reassessed. While no universally accepted definition exists regarding the time threshold for determining inadequate efficacy, current literature indicates that non-responders can often be identified within this timeframe [69]. Based on both evidence and clinical consensus, the period of 4 weeks can be considered a reasonable and pragmatic interval to evaluate treatment effectiveness in routine practice. In cases of inadequate response, the following options should be considered:

- Discontinuation of the PAMORA, particularly if no benefit is observed and side effects emerge.
- Combination therapy, wherein a PAMORA is continued alongside appropriately selected anti-constipation agents (e.g., stimulant or osmotic laxatives), especially if not previously initiated.
- Switching the opioid type or route of administration, which may help to re-establish a more favourable balance between analgesic benefit and gastrointestinal tolerability.
- Referral to a specialist (e.g., gastroenterologist, pain specialist, or internist) within a dedicated referral center, for further diagnostic work-up and individualized treatment planning.

Among these, the addition of a laxative—if not yet prescribed—is the most immediate and accessible intervention. Laxative type and dosage should be carefully tailored to the patient's symptom profile, comorbidities and tolerance.

Opioid rotation also represents a potentially effective strategy. Clinical experience and limited observational data suggest that switching to an alternative opioid (e.g., from morphine to tapentadol or transdermal fentanyl) may alleviate OIC symptoms while maintaining analgesia [70, 71]. However, this approach should be implemented cautiously and ideally under the supervision of a specialist, given the individualized pharmacodynamics and side-effect profiles of different opioid formulations.

Finally, in patients with refractory OIC despite guideline-concordant therapy and treatment adjustments, a specialist referral is warranted. Access to multidisciplinary expertise within referral centers, including internal medicine, gastroenterology, and pain management, can facilitate advanced and tailored interventions, ensuring comprehensive evaluation. This step aligns with the ECHO–OIC simplified care pathway and supports continuity of care for complex cases [26].

## Conclusion

OIC is among the most frequent and persistent adverse effects of chronic opioid therapy. Unlike other opioid-related side effects, OIC rarely resolves with continued treatment, with negative effects on patients’ quality of life and functional status, and on healthcare resource use. Despite the availability of valuable international guidelines and therapeutic options, OIC remains widely underdiagnosed and undertreated, especially in non-specialist settings [6, 15–17, 28, 72–87]. In response to this critical gap, the ECHO OIC European Committee recently introduced a simplified, evidence-based care pathway, explicitly developed to support primary care practitioners in the early recognition and structured management of OIC [26, 27]. A further step included the consensus by a multidisciplinary panel of experts to adapt and refine the European recommendations for use within the Italian healthcare context [30]. There is an urgent need to expand awareness of OIC across the broader healthcare community. Increasing clinician knowledge is essential not only to facilitate early diagnosis and effective intervention but also to support proactive prevention of OIC from the outset of opioid therapy. The role of primary care providers is particularly critical, given their frontline responsibility for managing chronic non-cancer pain, where OIC is often overlooked or minimized. Building upon the European framework and integrating practical insights from Italian clinical experience, the panel formulated a seven-step diagnostic–therapeutic pathway (Table 2). This structured approach is designed to guide timely and appropriate management of OIC in both primary and secondary care, with particular emphasis on education, early intervention, monitoring, tailored pharmacologic strategies, and coordinated referral when necessary.

**Table 2:** Seven-step clinical pathway for effective management of OIC

1. Inform patients at opioid initiation	Every patient prescribed long-term opioid therapy should be clearly informed about the high likelihood of developing constipation. Clinicians should explain that OIC is a common and expected consequence, not just a minor side effect. Educating patients early helps to avoid unnecessary opioid discontinuation and ensures prompt reporting of symptoms
2. Prescribe preventive laxatives alongside opioids	At the time of opioid initiation, physicians should co-prescribe a suitable laxative—preferably an osmotic agent, such as macrogol or a stimulant laxative. This approach aims to reduce the risk of constipation from the outset, especially in vulnerable groups such as elderly or cancer patients
3. Evaluate bowel function within 2 weeks	Early assessment is essential. Patients should be evaluated within 14 days of starting opioids, using detailed clinical history and established diagnostic criteria such as the Rome IV guidelines. Tracking stool form and frequency allows for early identification and classification of OIC
4. Consider peripherally acting µ-opioid receptor antagonist (PAMORAs) if laxatives fail	If constipation persists despite adequate use of first-line laxatives, a PAMORA should be considered, i.e., methylnaltrexone, naloxegol, and naldemedine, which are specifically designed to reverse opioid action in the gut without affecting analgesia
5. Use with caution: review drug labels and educate patients	When prescribing PAMORAs, physicians must review potential drug interactions and instruct patients on proper dosing and administration. Patients should understand that PAMORAs are taken daily, not as needed, and should not be stopped without medical advice
6. Monitor efficacy with the bowel function index (BFI)	Treatment effectiveness should be regularly monitored by the validated BFI, a simple tool assessing ease of defecation, stool completeness, and personal perception of constipation. A BFI score ≥ 30 may signal the need for treatment escalation or adjustment
7. Reassess after 4 weeks of PAMORA therapy	If the response to PAMORA treatment is insufficient after four weeks, several options should be explored: discontinuing the drug, combining it with other anti-constipation agents, switching the opioid class or route, or referring the patient to a specialist for further evaluation

Adapted from Varrassi et al. [30]

By aligning international guidance with local practice realities, this expert opinion provides a pragmatic and scalable model to improve outcomes for patients affected by OIC, and to strengthen continuity of care within Italy’s evolving pain management landscape. Key messages must be retained by the clinical community when handling issues related to OIC (Table 3).

**Table 3:** Key messages in the case of opioid-induced constipation (OIC)

1	OIC is one of the most frequent gastrointestinal adverse effects associated with chronic opioid therapy either in cancer patients or patients with chronic pain
2	Despite its high prevalence, OIC remains significantly underdiagnosed and insufficiently treated in clinical practice, with impact on quality of life of affected patients
3	To prevent such a burdensome side effect, clinicians must be aware of the relevance of OIC and must adopt effective preventive and curative measures for the management of OIC both at a primary and secondary care settings
4	In patients experiencing persistent constipation despite appropriate use of laxatives, clinicians should consider initiating treatment with a peripherally acting µ-opioid receptor antagonist (PAMORA) to address the underlying pathophysiology of OIC and restore bowel function without compromising analgesia

## Data Availability

Not applicable.
